# The Use of NIR Spectroscopy and Chemometrics to Identify the Thermal Treatment of Milk in Fiore Sardo PDO Cheese to Detect Fraud

**DOI:** 10.3390/foods14132288

**Published:** 2025-06-27

**Authors:** Marco Caredda, Alessio Silvio Dedola, Massimo Pes, Margherita Addis

**Affiliations:** Agris Sardegna, Servizio Ricerca Prodotti di Origine Animale, Loc. Bonassai, 07100 Sassari, Italy; adedola@agrisricerca.it (A.S.D.); mpes@agrisricerca.it (M.P.); maddis@agrisricerca.it (M.A.)

**Keywords:** Fiore Sardo PDO, NIR spectroscopy, milk thermal treatment, linear discriminant analysis, genetic algorithms, sheep cheese

## Abstract

The production of Fiore Sardo cheese is regulated by the specification of the Protected Designation of Origin (PDO), which aims to guarantee the specific area of production, the know-how of local producers, and the specific use of raw milk from Sarda sheep. The thermization of milk is a sub-pasteurization process that is commonly used in cheese-making to lower the bacterial load and increase the shelf life of the product; it is therefore a cause of non-compliance with the PDO specification of Fiore Sardo cheese, allowing producers to gain practical and economic advantages. In this work, NIR spectroscopy coupled with multivariate discriminant analysis was used to identify the thermal treatment of milk in Fiore Sardo cheese samples. Cheeses were produced using raw milk (38 °C), low-thermized milk (57 °C for 30 s), and high-thermized milk (68 °C for 30 s). The NIR spectra of the cheeses were used to build discriminant models for individuating the thermal treatment of the processed milk. The obtained discriminant models were able to correctly classify about 90% of the Fiore Sardo cheese samples. This method could be suitable as a screening technique to authenticate Fiore Sardo PDO cheese.

## 1. Introduction

Raw milk from Sarda sheep is used for the production of one of the most ancient and traditional Sardinian Protected Designation of Origin (PDO) cheeses—Fiore Sardo. Raw milk contains enzymes, as well as a microbial flora that is composed of a variety of bacteria, yeast, and molds that can play an important role in the formation of different and distinctive compounds during the aging of dairy products. The use of raw milk is essential to retain all the intense and unmistakable nutritional and sensory characteristics of cheese, strongly connecting it to the use, in livestock feeding, of pastures of the production area. The production of Fiore Sardo cheese is regulated by the PDO specification (Reg CE n. 1263/96), which implies the use of raw ovine milk.

On the other hand, raw milk has to be managed with care, reducing its storage time, as it contains undesirable microorganisms that could grow even at refrigerating temperatures. Fermentative issues in dairy products and the risk for the consumers’ health are the most frequent unwanted consequences. For these reasons, milk is generally thermally treated in the dairy industry with the aim of reducing the levels of bacteria, viruses, molds, yeasts, and protozoa, thus achieving the high requirements of food safety and extending the shelf life of the products.

As concerns cheese production in Sardinia, for sheep-milk cheeses other than Fiore Sardo PDO, the most used thermal treatment of milk is thermization. Milk thermization consists of a sub-pasteurization process at 57–68 °C (Directive 92/46/EEC) for 15–30 s that, if applied during the production of Fiore Sardo PDO, represents a serious fraud that is punishable with administrative and criminal penalties, as well as with the revocation of PDO certification.

Whereas small artisanal producers can work the raw milk of their own production within a few hours from milking, thereby reducing the risk of bacterial proliferation, bigger dairy industries collect raw milk from several producers that are located in different areas of the island, thus lengthening transport times and consequently increasing the risk of contamination and bacterial proliferation in milk. Due to these reasons, bigger dairy plants must adopt separate production lines for raw milk and for thermized milk in order to avoid cross-contamination. However, the application of milk thermization can be an easy shortcut, allowing producers to avoid both the separate collection of milk and the implementation of separate processing lines. Undoubtedly, this impacts on the economy and daily routine of the farm, and it should be taken into consideration that the amount of raw milk processed in dairies is much less than that of thermized milk. Thus, milk thermization is the most common cause of non-compliance with the PDO specification of Fiore Sardo cheese.

Although it is undeniable that heat treatment could allow us to avoid all the issues related to the use of raw milk and consequently extend the shelf life of the product, milk thermization causes a detrimental effect on the nutritional and sensory properties of the produced cheese, determining a loss of flavor and aroma, as well as damaging the reputation of the product. In fact, as reported by other authors, the thermization of processed milk causes a marked decrease in the level of lipolysis in Fiore Sardo cheese—in particular after 180 days of ripening—compared to what happens in cheese made from raw milk; instead, the proteolysis is not affected by the thermal treatment in a significant way, as it is only present at a low level in this type of cheese [1,2,3]. It is therefore necessary to develop a method for detecting the presence of the thermal treatment of milk in Fiore Sardo PDO cheese.

The thermal treatment of milk causes changes in the chemical composition of dairy products, such as the volatile compound profile [3,4,5], the polar metabolite profile [3], and the casein micelle structure [6]. Moreover, endogenous enzymes such as alkaline phosphatase (ALP, EC 3.1.3.1), lactoperoxidase (EC 1.11.1.7), and γ-glutamyltransferase (GGT, EC 2.3.2.2) present a lower activity after the thermization of milk. The inactivation of these enzymes is usually determined to assess the presence of a thermal treatment for safety purposes [7], but their inactivation temperature (~72 °C) is too high with respect to the temperatures used for the sub-pasteurization of milk (57 °C to 68 °C), which is normally practiced in local dairy factories. For this reason, our research group had studied the presence of another endogenous thermolable enzyme in ovine milk—α-L-fucosidase (EC 3.2.1.51) [8]. We determined that its inactivation occurs after heating at temperatures between 57 °C and 68 °C [9]. Therefore, an RP-HPLC method has been developed to evaluate α-L-fucosidase enzyme activity in Fiore Sardo PDO cheese to determine if the milk used in the cheese-making process has been thermally treated or not [10]. Despite the high sensitivity of the method, its application to the routine control of PDO cheeses is not affordable as it requires the use of chemicals, chromatographic instrumentation, and skilled operators, thus being expensive and time consuming. Spectroscopic techniques have the advantage of being non-destructive, inexpensive, solvent-free, and rapid; they could be a valid alternative to chromatographic methods.

The chemical changes induced by the thermal treatment of milk result in the modification of dairy product spectra acquired using different spectroscopic techniques, such as visible [11], infrared [11], nuclear magnetic resonance (NMR) [11,12,13], and fluorescence [14] spectroscopies. For discriminant purposes, Raman was used on cow milk samples to individuate the thermal treatment they had been subjected to, taking into consideration a pasteurization temperature of 65 °C for 30 min [15]. Anedda et al. [16] evaluated the thermal treatment of ovine milk by directly analyzing Fiore Sardo cheese by means of magnetic resonance imaging (MRI) and image analysis.

To the best of our knowledge, NIR spectroscopy has never been used in the identification of the thermal treatment of milk in dairy products. Nonetheless, NIR spectroscopy has been widely used in the quality control of cheeses thanks to the possibility of combining it with multivariate analysis. In fact, NIR spectra can be associated with a particular property or characteristic (quantitative or qualitative), with the aim of developing prediction models for estimating the content of chemical compounds in the matrix or for classifying the samples based on their characteristics. In addition to the quantitative estimation of various chemical components in dairy products [17,18,19,20,21,22,23], which is the common use of NIR spectroscopy, this technique was studied in combination with multivariate discriminant algorithms for authentication purposes. Among the works found in the literature, NIR was evaluated in relation to the tracing of the geographical production origin of cheese [24,25,26,27]; in the identification of the animal origin of raw milk (bovine, caprine, or ovine) in cheese [28]; in the classification of cheese samples concerning their ripening age [29,30], their period of production, and the location of the cheese factories [29]; and in the classification of cheese based on the feed given to the dairy cattle (pasture or preserved forage) [31].

For fraud control purposes, NIR was investigated to detect the presence of adulterants in cheeses; these are usually used for economic gain and can lead to health concerns due to false or misleading statements made about a product [32,33,34]. NIR was used to detect the addition of vegetable oil in cheese [35] and butter cheese [36], as well as to detect the presence of cow milk in goat-milk cheeses [37,38] and in goat-milk yogurt [38]. Alinovi et al. [39] and Calvini et al. [40] used NIR spectroscopy to determine the presence of different percentages of rind in grated Parmigiano Reggiano cheeses. For a deeper insight into the methods used to detect fraud in dairy products, including NIR spectroscopy, the reader may refer to the recent reviews by Hebling e Tavares et al. [41] and Abedini et al. [42].

Seeing the potential of NIR spectroscopy, this study evaluated the ability of NIR combined with chemometrics to identify if the milk used to produce Fiore Sardo PDO cheese is subjected to a thermal treatment. Two different temperatures of milk treatment (57 °C and 68 °C) were considered. Genetic algorithms (GAs) [43,44,45,46] were applied to the NIR spectra of the cheeses to select the informative wavelengths. The use of GAs was assessed by our research group by applying them to the Fourier Transform Mid Infrared (FT-MIR) spectra of dairy and beehive products in order to build reliable and robust models to predict the composition [47] and the characteristics [48,49] of sheep milk, to determine the botanical origin of unifloral honeys [50], and by applying them to the NIR spectra of honey samples in order to discriminate their geographical and botanical origin [51].

The identification of the thermal treatment of milk by directly analyzing cheese is more challenging than detecting it in milk. In fact, some milk components that are changed due to thermal treatment (such as the polar fraction) are lost in the cheese-making process. Nonetheless, the fraud control of Fiore Sardo PDO cheeses has to be carried out on samples that are already labeled with the PDO certification. As described above, NIR spectroscopy instrumentation has the advantage of being commonly used in the routine analysis of cheeses by different laboratories; therefore, the method developed in this work could be easily transferred to the NIR instrumentation of local laboratories. The identification of the thermal treatment of milk in Fiore Sardo PDO cheese using NIR spectroscopy could become a screening method in the authentication analysis of this type of cheese. This makes NIR spectroscopy the preferred technique in this context.

## 2. Materials and Methods

### 2.1. Cheese-Making Procedure

In the period between January and June 2019, 18 cheese-making trials—3 per month—were performed in the experimental cheese factory of the Agenzia Agris Sardegna. Raw whole sheep milk from the Sarda breed flock of Agris’ experimental farm was used. In each trial, 105 kg of refrigerated raw milk from a single batch was split into three aliquots of 35 kg; the first one was heated at 38 °C (raw milk—RM), the second was heated at 57 °C for 30 s and quickly cooled down to 38 °C (low-thermized milk—LTM), and the third was heated at 68 °C for 30 s and quickly cooled down to 38 °C (high-thermized milk—HTM). The heating of RM and the thermal treatments of LTM and HTM were performed in a batch-wise (discontinuous) process using an infrared tubular heat exchanger capable of rapid cooling (model Stoutz-Actinator, Actini Group, Évian-les-Bains, France). Each aliquot of milk was then subjected to the cheese-making. The Fiore Sardo PDO cheese type was manufactured for analytical purposes. In order to eliminate any possible differences resulting from the cheese vat used, three different single-walled cheese-making vats were alternatively used in the cheese-making process of RM, LTM, and HTM during the cheese-making trials. However, the dairyman remained the same. During each cheese-making trial, cheese milk (RM, LTM, and HTM) was inoculated with a freeze-dried starter culture composed of mesophilic lactic acid bacteria species (CHOOZIT MA11 LYO 50 DCU, Danisco, Kopenhagen, Denmark) at 7 Direct Culture Unit (DCU) 100 kg^−1^ of milk. After a few minutes, a soluble lamb rennet paste (140 IMCU·g^−1^; Caglificio Manca, Thiesi, Italy), dissolved in a deionized water solution (1:10, *w*/*w*), was added at a concentration of 6500 IMCU·100 kg^−1^ of milk, allowing the milk to coagulate within 12 ± 1 min. After 1.5 times the clotting time, which was needed to allow for firming to occur, the coagulum was manually cut to obtain curd grains of about 2–4 mm. Curd was then introduced directly into perforated cylindrical molds for whey drainage and cheese wheel molding. Cheese wheels were transferred to the sweating room at 34–36 °C for 3–4 h until they reached a predetermined pH value of end acidification (5.31 ± 0.04 UpH). The obtained cheeses were categorized as raw milk cheeses (RCs), low-thermized-milk cheeses (LTCs), and high-thermized-milk cheeses (HTCs). Each cheese-making vat produced 5.8 ± 0.4 kg, producing three cheese wheels with individual weights of 1.9 ± 0.1 kg. The cheeses were then salted in brine (22–24% of NaCl) for 12 h kg^−1^ cheese, before being ripened at 12 ± 2 °C and a relative humidity of 80 ± 5% for up to 180 days. A total of 36 RCs, 36 LTCs, and 36 HTCs (105 and 180 days of ripening) were analyzed.

### 2.2. Near-Infrared Spectra Acquisition

NIR spectra were acquired at 25 °C using a NIRSystem 5000 (FOSS, Padova, Italy) NIR spectrometer. Spectra were recorded on grated cheese samples, after sampling the cheese wheels by removing the rind. Spectral acquisition was made in reflectance (R) mode, in the range between 1100 nm and 2498 nm, with 32 scans. Each spectrum, consisting of 700 points (resolution = 2 nm) and expressed as log(1/R), was obtained as an average of the 32 scans. Spectrometer performance was routinely assessed by checking repeatability and wavelength accuracy.

### 2.3. Chemometric Analysis and Genetic Algorithms

Principal component analysis (PCA) was performed for data visualization and outlier removal, whereas linear discriminant analysis (LDA) was used for building the classification models. Prior to the building of the models, genetic algorithms (GAs) were used to select the informative spectral regions to be used as predictors in LDA. This was a necessary step as LDA cannot be run when the ratio of the number of variables over the number of samples is high and when variables are highly correlated. PCA was performed using the R-based software Chemometric Agile Tool (CAT) developed by the Italian group of Chemometrics, Genova, Italy [52], while GAs and LDA were run on MATLAB R2021a release (Torino, Italy). All the performed elaborations were carried out on autoscaled data. The parameters set for GA variable selection were as follows: (1) maximum number of x variables: 200; (2) population size: 30 chromosomes (on average, five variables per chromosome in the original population); (3) discriminant method: LDA; (4) response: cross-validated accuracy (five deletion groups); (5) maximum number of variables selected in the same chromosome: 30; (6) probability of mutation: 1%; (7) number of runs: 100; (8) backward elimination after every 100th evaluation and at the end (if the number of evaluations is not a multiple of 100); (9) window size for smoothing: 3; (10) pretreatment: none; (11) scaling: autoscaling.

In order to find the best LDA model, the use of raw spectra, of standard normal variate (SNV)-transformed spectra, and of multiplicative scatter correction (MSC)-transformed spectra were evaluated; each of these pretreatments was also evaluated by applying first derivative and second derivative functions, for a total of nine different LDA models. Each LDA model was built performing a cross-validation (CV) with five deletion groups (5-fold CV). Stating that TP = true positive, TN = true negative, FP = false positive, and FN = false negative, the LDA confusion matrixes were evaluated in terms of (a) sensitivity (true positive rate) = TP/(TP + FN); (b) specificity (true negative rate) = TN/(TN + FP); (c) accuracy, calculated as the average of the percentages of correct predictions of each category; and (d) F1 score = 2 × [(p × s)/(p + s)], where p (precision) is TP/(TP + FP) and s is sensitivity.

### 2.4. Sample Partitioning

The 108 Fiore Sardo samples (105 and 180 days of ripening) belonged to three thermal treatment categories (RC, LTC, and HTC); however, to verify the capacity of NIR spectra combined with LDA in discriminating the samples in order to improve accuracy, different LDA models based on different categories were built. Thus, LDA models for the following comparisons were evaluated: (a) RC vs. LTC vs. HTC; (b) RC vs. LTC; (c) RC vs. HTC; (d) RC vs. TC (TC includes LTC and HTC samples); and (e) LTC vs. HTC. The chemometric elaborations concerning the LDA modeling described in Section 2.3 were applied to each of the comparisons here described.

## 3. Results

### 3.1. Analysis of NIR Spectra

An NIR spectrum (700–2500 nm) arises from the combination bands and overtones of the C-H, N-H, and O-H bond absorptions; NIR bands usually overlap, and the peaks are broad and of difficult interpretation with respect to those recorded in the middle infrared region. The third overtone absorptions lie in the spectral region between 700 and 1100 nm; absorptions due to the second overtone of the chemical bonds characterize the region between 1000 and 1550 nm; the region between 1550 and 2000 nm contains bands related to the first overtone absorptions, while bands in the region from 2000 to 2500 nm are due to the combination bands of two or more simultaneous vibrations [23,53,54,55,56]. In particular, O-H and N-H stretching–bending combinations lie near 2000 nm, whereas C-H combination bands occur between 2000 and 2400 nm; O-H and N-H first overtone absorptions lie near 1400 nm and 1500 nm, respectively, whereas C-H absorptions occur between 1600 and 1800 nm. The second overtone absorptions of C-H bonds occur between 1100 and 1300 nm. The second overtone vibration of O-H and N-H bonds, as well as the third overtone absorptions of all chemical bonds, were not recorded in the spectra analyzed in this study (spectral range: 1100 to 2498 nm) as they lie in the region between 700 and 1100 nm [57].

**Figure 1:**
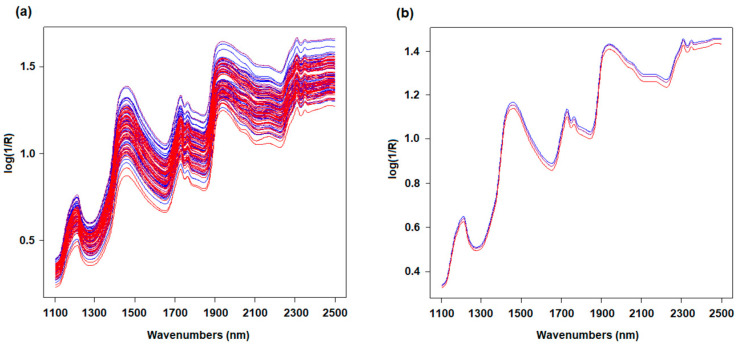
(**a**) NIR spectra of the 108 cheese samples. (**b**) Mean spectrum for each category of cheese. Blue = RC; purple = LTC; red = HTC.

Figure 1a shows the overlapping of the NIR spectra of the 108 Fiore Sardo samples, while Figure 1b plots the mean spectrum of each category. The different colors correspond to the three cheese categories—RC samples are plotted in blue, LTC samples are plotted in purple, and HTC samples are shown in red. The separation of the single cheese spectra in relation to the thermal treatment was not visible; even looking at the mean spectra, only a small difference is present among the samples, with the HTC mean spectrum slightly differing from the others.

### 3.2. Principal Component Analysis

**Figure 2:**
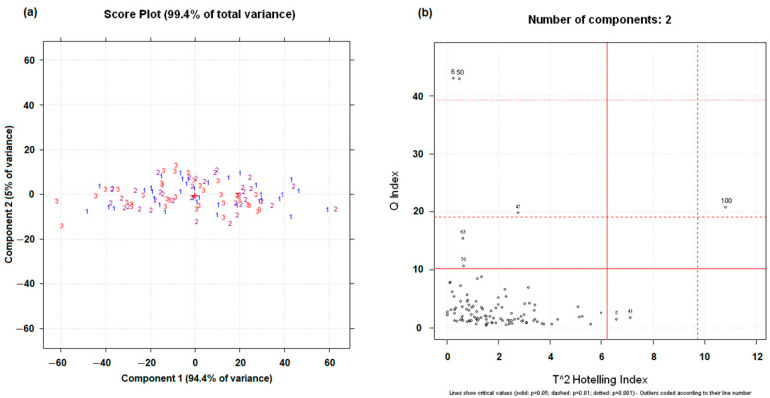
Score plot obtained on the first two principal components (**a**). T^2^ vs. Q diagnostic plot (**b**). Samples are numbered and colored based on their thermal treatment (1—blue = RC; 2—purple = LTC; 3—red = HTC).

The spectral dataset was subjected to principal component analysis in order to visualize the possible separations of the categories of samples and to individuate outliers. Figure 2a shows the score plot obtained by the first two principal components, describing 99.4% of the total variance. The samples, numbered and colored according to their thermal treatment category (1—blue = RC; 2—purple = LTC; 3—red = HTC), are spread in the plot without category separation. Even when plotting other principal components, a separation was not achieved. PCA was repeated after applying the spectral transformations described in Section 2.3; however, none of these treatments led to an improvement in the separation of the samples in relation to the thermal treatment applied. The T^2^ vs. Q diagnostic plot obtained for two principal components (Figure 2b) was used to verify the presence of outliers. Samples 6, 50, and 100 were considered as outliers and were removed from the dataset, thus resulting in a total of 105 samples (35 samples for each of the three thermal treatment categories).

### 3.3. Building of LDA Models

Linear discriminant analysis (LDA) was performed on Fiore Sardo NIR spectra to classify the samples on the basis of the thermal treatment that had been applied on the processed milk. Since LDA cannot be run on whole spectra, due to the large number of variables and their correlation, GAs were applied to select the informative spectral variables able to discriminate the samples. GAs are based on the concept of the Darwinian evolution of the species, whereby individuals with a greater adaptability to the environment have a greater probability of surviving and reproducing. The best individuals will transmit their genetic content to the following generations, while the genetic content of the worst individuals will be lost. In spectral data, an individual is described by a combination of spectral variables (chromosome) in which a single variable (gene) is coded with 0 if not selected or with 1 if selected. GAs create a population of individuals and evaluate their adaptability to the environment (the accuracy in cross-validated LDA). According to this response, the best individuals are most likely chosen to reproduce and generate offspring through a process of cross-over and mutation. GAs evaluate the new chromosomes and if their response is better than that of the worst chromosomes of the population, they will replace them. This process is repeated several times, maintaining the number of individuals of the population as constant. Each time, only the most informative chromosomes are kept, leading to an improvement in the average response of the population. Thus, only the informative variables will be maintained, whereas those not useful for the discrimination will be discarded. The performances of GAs are optimized when the number of starting variables is less than 200 [45]; therefore, the number of spectral wavelengths of the spectrum (i.e., 700) had to be reduced. In order to minimize a possible loss of information, three working options for reducing the number of variables were studied. The first involved reducing the 700 wavelengths to 175 through averaging over 4 adjacent wavelengths, and running GAs on the 175 new spectral variables; for each selected variable, all four variables of the original spectrum used in the averaging were considered. A second GA selection was carried out on the selected variables to reduce their number, finding the most informative ones. The second option involved dividing each spectrum into two portions of 350 variables each and reducing the 350 variables of each portion to 175 by averaging over 2 adjacent wavelengths. Then, GAs were run separately on the two portions each containing 175 variables. Additionally, for each of the selected variables, the two original variables used in the averaging were considered. The selected variables of the two portions were then collected and re-subjected to a selection in order to reduce their number to be used in LDA. The third option comprised dividing each spectrum into four portions of 175 variables each and running GAs separately to each of them (without the need for averaging). The selected variables of the four portions were then collected and re-subjected to a selection in order to find the most informative ones.

The three procedures elaborated on raw spectra were replicated after separately applying the following spectral pretreatments to the dataset: 1st derivative, 2nd derivative, SNV, SNV + 1st derivative, SNV + 2nd derivative, MSC, MSC + 1st derivative, and MSC + 2nd derivative.

Table 1 shows the results (expressed in the form of the accuracies in cross-validation) obtained using the LDA models built to discriminate the Fiore Sardo cheese samples concerning the thermal treatment of the processed milk. The table reports the different types of sample comparisons (column “categories to be discriminated”) and for each of them, the results obtained using the LDA models built using different mathematical transformations of the spectra. The three columns indicating the accuracies refer to the three working options used to select the informative variables for the discriminations.

As can be seen, there is a general trend in the accuracies when comparing the three methods of reduction in the spectra to obtain a number of starting variables for GA selection lower than 200, whereby the results generally became better with lower levels of reduction in the spectral variables. The GA selection carried out on four-averaged variable spectra generally led to the worst results of the LDA models, whereas the one carried out on the original spectra led to the best results. Intermediate results were obtained when applying GAs to the two-averaged variable spectra. This is valid for all the elaborations carried out, with very few exceptions, such as the models built to discriminate LTC and HTC samples, with the highest accuracy being obtained by running the GA in the intermediate working option. Since the best results were achieved in the working option that considered the non-averaged spectra, the following sections will be based only on these results, with the exception of the LTC vs. HTC comparison.

#### 3.3.1. Linear Discriminant Analysis to Discriminate RC, LTC, and HTC Samples

In the discrimination of the samples relating to the three different thermal treatments of milk, the application of different spectral pretreatments resulted in LDA models being characterized by accuracies ranging from 59% when using first derivative-transformed spectra to 76% when using second derivative SNV-transformed spectra (Table 1, last column). The confusion matrix obtained using the best model is shown in Table 2, together with the informative spectral regions selected by the GA. In total, 27 RC samples were correctly classified (sensitivity = 0.771), 5 RC samples were misclassified as LTC samples, and 3 RC samples were misclassified as HTC samples. A similar performance (sensitivity 0.714) was obtained for the samples in the LTC category, with 25 of them being correctly classified, 4 being misclassified as RC samples, and 6 being misclassified as HTC samples. The HTC sample category was the most correctly predicted, with 28 samples out of 35 (sensitivity of 0.800). However, 2 samples were misclassified as belonging to the RC category, while 5 samples were identified as LTC samples. The specificity ranged between 0.857 and 0.914, indicating the capacity of the model to avoid false negatives. F1 scores, ideally close to 1, were between 0.714 and 0.794.

#### 3.3.2. Linear Discriminant Analysis to Discriminate RC and LTC Samples

The categories of the raw milk cheese samples and of the low-thermized-milk cheese samples were discriminated using the models built with raw, SNV, and MSC spectra with similar accuracies (73%, 77%, and 77%, respectively). The first and second derivative applied to the different pretreatments improved the results, with the highest accuracy obtained using the second derivative raw spectra (93%). This model misclassified only 1 RC sample out of 35, as well as 4 LTC samples out of 35, with values of sensitivity 0.971 and 0.886, and of specificity of 0.886 and 0.971 for the two categories of samples. The F1 scores (0.931 and 0.925 for the two categories of samples) indicate that the prediction is well balanced (Table 2).

#### 3.3.3. Linear Discriminant Analysis to Discriminate RC and HTC Samples

RC samples and HTC samples were distinguished using the LDA models based on the not-treated, SNV, and MSC spectra, with accuracies being close to 80%. The first and second derivative transformations increased the accuracies of the models reaching the highest result (91%) by elaborating the spectral regions selected on the 2nd derivative MSC spectra (Table 1). The corresponding confusion matrix (Table 2) shows that the two categories were balanced in the classification, with 32 out of 35 samples being correctly assigned for both groups. Since the confusion matrix is symmetrical, the sensitivity, specificity and F1 scores have the same value (0.914), indicating the very good modeling of the samples.

#### 3.3.4. Linear Discriminant Analysis to Discriminate RC and TC Samples

The classification of RC and TC samples was between 74% and 79% when modeling raw, SNV, and MSC spectra. The 1st derivative transformation led to models with accuracies ranging from 79% to 80%, but it was the 2nd derivative that improved the models, with the best accuracy being obtained with the regions selected on the 2nd derivative raw spectra (89%) (Table 1). The sensitivity and specificity of the single categories were the same (0.886), while the F1 scores were 0.838 and 0.912 for the two categories, with a good balance in the classification.

#### 3.3.5. Linear Discriminant Analysis to Discriminate LTC and HTC Samples

The classification of LTC and HTC samples was obtained using the raw-, SNV-, and MSC-based spectra models, with accuracies ranging from 69% to 80%. When the 1st derivative was applied to the spectra (raw and transformed), it improved or maintained similar results (79% to 84%), whereas the 2nd derivative led to accuracies ranging from 84% to 86%. Nonetheless, the best result was not among these models, which were obtained by selecting the spectral variables by dividing the spectra into four portions (Table 1, last column), even if these results were generally the best ones. In fact, an accuracy of 90% was obtained using the 2nd derivative MSC spectra-based model built with the spectral variables being selected by dividing the spectra in two portions (averaging over two adjacent variables). The confusion matrix (Table 2) shows that 31 out of 35 LTC samples and 32 out of 35 HTC samples were correctly classified. The true positive and true negative rates are high for both categories (0.886 and 0.914, as well as 0.914 and 0.886, respectively), with very similar F1 scores (0.899 and 0.901, respectively).

#### 3.3.6. Misclassified Samples

Table 3 shows which samples were misclassified considering all the five best models and the thermal treatment categories. As seen in the previous paragraphs, the model built to discriminate RC, LTC, and HTC samples together was the one that misclassified a higher number of samples, whereas the other models had similar accuracies, close to 90%. Without considering the model “RC vs. LTC vs. HTC”, as well as taking into account the three models containing the RC samples (“RC vs. TC”, “RC vs. LTC”, and “RC vs. HTC”), it can be seen that most of the wrongly assigned samples were misclassified by only one of the three models. Instead, four samples (i.e., 25, 48, 55, and 71) were not correctly identified by two of the models, whereas none of the samples were misclassified by all three models.

## 4. Discussion

The identification of a mild milk thermal treatment in Fiore Sardo PDO cheese is of great interest for assessing the presence of fraud. In fact, the production of this type of cheese is regulated by the PDO specification that implies the use of raw ovine milk. Instead, the thermization (or sub-pasteurization) of milk, which involves a mild heat treatment consisting of heating milk at 57–68 °C, is a practice that aims to eliminate pathogenic bacteria and reduce spoilage bacteria in milk, enhancing cheese safety and storability, minimizing fermentation defects, reducing production losses, and extending the cheese’s shelf life. Milk thermization is automated in industrial dairies and is commonly used in the production of several Sardinian cheeses other than Fiore Sardo. The process is usually applied to milk for a minimum holding time of 15 s. However, the heating holding time is not defined in the Directive 92/46/EEC that states the conditions for performing milk thermization. Instead, the Directive requires that the enzyme alkaline phosphatase ALP in thermized milk must have a positive reaction. The inactivation of the alkaline phosphatase enzyme depends on both the temperature and holding time of heating; it was found that it is completely inactivated when milk is heated at 63 °C for 30 min or when heated at 72 °C for 15 s [58]. It is therefore clear that the ALP method cannot be employed to detect if a mild thermal treatment (sub-pasteurization) is applied to milk.

Our research group focused on another thermolable enzyme in ovine milk—α-L-fucosidase—which is inactivated at temperatures between 57 °C and 68 °C. We developed a method based on RP-HPLC instrumentation to detect this enzyme [10]. Cheeses were obtained both using raw milk (38 °C), sampled along the ovine lactation period (December to May), and using the corresponding thermized milk (68 °C for 30 s). The thermal treatment caused a lowering of the activity of this enzyme to about one-third of the original activity, but a dependence of α-L-fucosidase activity on the lactation period was found. Therefore, this method cannot be employed in routine controls, as it is expensive and time-consuming.

Therefore, the aim of this work was to assess a fast and cheap method to be used as a form of screening in the fraud control of Fiore Sardo PDO cheese. NIR spectroscopy is widely known to be a rapid, economic, and nondestructive method and in this work, it was employed for the first time on cheese samples to identify the thermal treatment of the milk used in the cheese-making process.

The results show that NIR coupled with chemometrics was able to correctly individuate the thermal treatment of most of the Fiore Sardo cheese samples. The best discriminant models required the investigation of some operations such as the division of the spectra into different portions to be subjected to GA wavelength selection, as well as the pre-processing of the spectra with different mathematical transformations. Since GAs perform better when running on no more than 200 variables, a reduction in the number of variables was needed. The simplest and fastest way of carrying this out was to average the spectra over four adjacent wavelengths, obtaining “new” spectra of 175 variables calculated from the starting 700 ones; however, the results obtained were not always satisfactory. Averaging over two adjacent wavelengths resulted in a general improvement in the accuracies, but it was the elaboration of not-averaged spectra that led to the majority of the best results. This is a clue that the averaging operation caused a loss of information.

Concerning the different spectral pretreatments, the 2nd derivative was the one that led to most of the best results, both when applied to raw spectra and when applied to SNV- or MSC-transformed spectra, together with the 1st derivative that led to one of the best results (RC vs. HTC). Having broad peaks, NIR spectra usually need to be transformed before their elaboration, e.g., the 2nd derivative is used for resolution enhancement and baseline correction, removing offset and background drifts due to scattering, better resolving some spectral bands that were originally overlapping [23].

The choice of performing models for different comparisons of thermal treatment categories of the samples was carried out with the aim of improving the accuracies obtained. In fact, the elaboration performed by considering all three categories of thermal treatment (RC vs. LTC vs. HTC) was the most challenging and, as expected, the accuracy was the lowest obtained (76%), with the intermediate group (LTC) resulted in the most misclassified samples. Including LTC and HTC samples in one category (TC) led to an improvement (89%) in the number of correctly classified raw and thermized samples. Moreover, when comparing RC samples separately with LTC and HTC samples, the models were able to correctly individuate a higher number of samples; the obtained accuracies were very similar among the two models (93% and 91%, respectively), even if a slightly better classification of RC samples was obtained in comparison to LTCs. All the classifications were balanced, considering the different categories of samples; this can be seen by the results of sensitivity, specificity, and F1 score, which are very similar among the different categories and, in most cases, are close to 1.000. The models are accurate and able to avoid false negatives and false positives. The last type of comparison consisted of the identification of only thermally treated cheese samples concerning the two temperatures of milk thermization (LTC (57 °C) vs. HTC (68 °C)). Additionally, this elaboration was considered challenging as it was seen that chemical changes due to milk thermal treatment, such as the inactivation of the enzyme α-L-fucosidase, occur at both temperatures. Nonetheless, the best model obtained was able to classify 90% of the samples, indicating that the different temperatures of thermization led to a change in the spectra profile.

**Figure 3:**
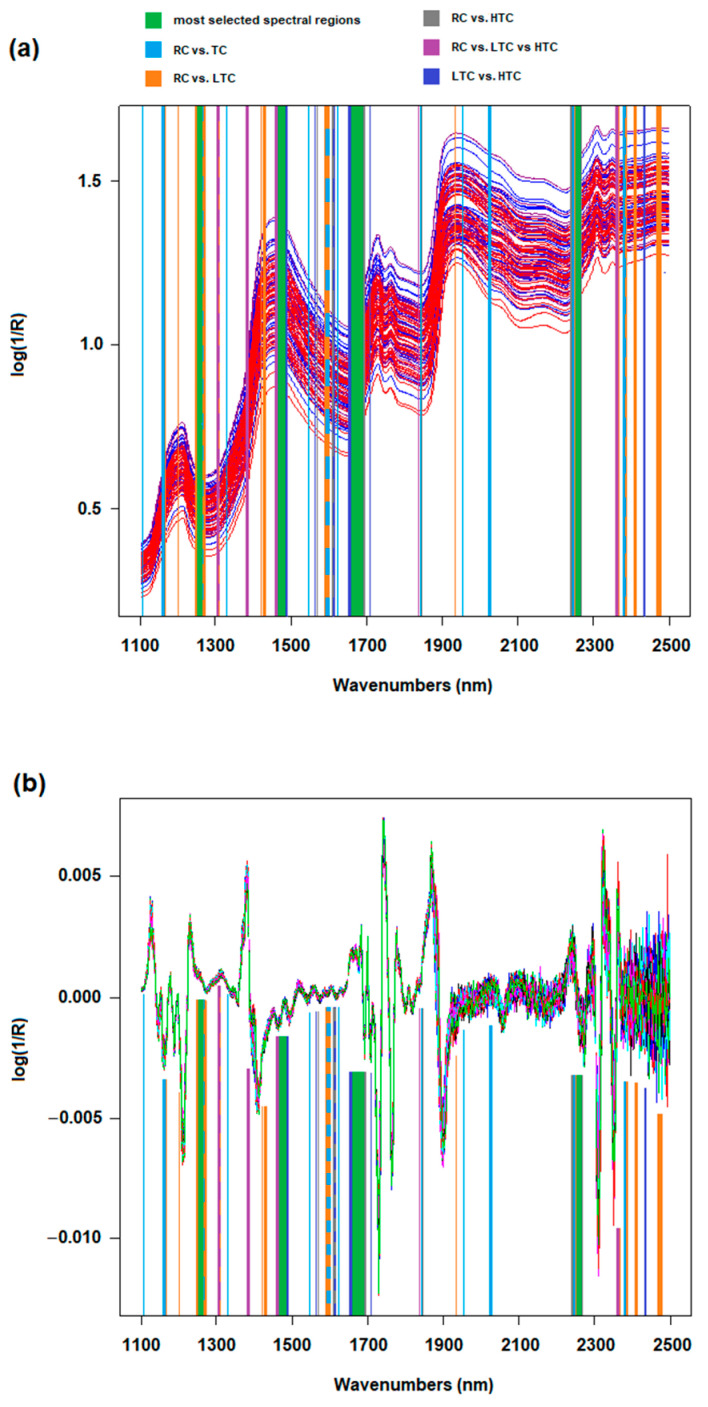
Selected spectral regions for all the best models obtained for each category comparison reported in the plot of (**a**) the not-pretreated spectra (cheeses samples are colored as: blue = RC; purple = LTC; red = HTC) and (**b**) the 2nd derivative SNV spectra.

At this point, the GA selected some common regions among the best models obtained for each category, such as 1256 to 1258 nm, 1470 to 1474 nm, 1676 to 1688 nm, and 2262 to 2264 nm (Figure 3, green lines); these contain most of the information related to the three thermal treatment categories, but are not sufficient to correctly classify the samples by themselves. In fact, the GA found other wavelengths or spectral regions, different for each type of comparison, to be important for the discrimination of the categories of cheese samples. Most of these variables are located in the regions from 1132 to 1700 nm and from 2356 to 2468 nm; they sometimes overlap and some of them were adjacent to the most selected common regions cited above (Figure 3a). In addition to some wavelengths around 1840 nm and 1950 nm, the region from 1700 to 2150 nm was not informative for the classification of the cheese samples. The difference in the selection of the wavelengths among the models could be related to the type of spectral pretreatment that the spectra were subjected to; however, it is more likely that the GA kept only some of the wavelengths in each model, rejecting the redundant ones. The spectral regions in common to all models contain absorptions due to the 2nd overtone of the C-H vibration, to the 1st overtone of the C-H and O-H vibrations, and to the combination bands of the C-H bond. The other spectral regions selected by the GA are not common to all the models and generally correspond to the same absorptions; however, only the models discriminating RC vs. HTC and RC vs. TC include regions related to the N-H functional group (1540 nm and 2152 to 2158 nm). These chemical functional groups are typical composed of different compounds in milk, related to fat, proteins, sugars, and enzymes, which could change their structure or their level when the milk is heated. Caboni et al. [3] found that a significant difference in the level of the short-chain free fatty acid in Fiore Sardo cheeses aged 105 days is present between those produced with raw and thermized milk, with higher levels in the raw milk cheeses; in 180-day-aged cheese, this difference was also found in relation to the medium- and long-chain free fatty acids; this could be explained by a partial inactivation of the endogenous lipoprotein lipase (LPL) when the milk is heated. The authors also determined the metabolomics profile of the cheeses, finding that, among others, galactose, gluconic acid, fucose, and serine were the most discriminating agents between the raw-milk cheeses and the thermized-milk cheeses. Changes in the spectral profiles are likely due to the combination of the changes in the levels of all these compounds, which are characterized by the same chemical bonds whose absorptions overlap. It also has to be said that GAs are able to find informative regions for specific scopes that are not theoretically bound to the absorption of any functional group and that a spectroscopist would never select [59]. This happened in some works in which GAs were used to select the informative regions of sheep milk and of honey FT-MIR spectra for different classification scopes, by developing models with wavelengths not directly related to an absorbance peak, e.g., the geographical traceability of milk was determined on a test set with a 99% accuracy LDA model [48]. The different type of pasture and the access time to the pasture of ewes were classified, validated with accuracies of 100% and 77% [49]; the botanical classification of honey was performed with an 88% accuracy on a test set [50]. In the work proposed in this paper, GAs were chosen as a selection method over other techniques for the reliability and stability they demonstrated in these studies. Even in this work, GAs selected some wavelengths that, as seen in Figure 3b, do not correspond to a peak even on 2nd derivative SNV-transformed spectra, but led to good classification accuracies. Therefore, GAs continue to demonstrate their effectiveness in finding areas related to the achievement of a specific goal.

Few studies were found in the literature that aimed to assess the thermal treatment of milk and dairy products using spectroscopic methods. In the work by Yazgan et al. [15], Raman spectroscopy and PLS-DA were used to discriminate raw and pasteurized milk samples, obtained by heating milk at 65 °C for a prolonged time of 30 min, with an accuracy close to 100%. Due to the importance of detecting fraud in Fiore Sardo PDO cheese, the only study aimed at the determination of milk’s thermal treatment in cheese was the study performed by Anedda et al. [16] in which artisanal and commercial Fiore Sardo PDO cheeses were analyzed using MRI relaxometry and image analysis; the authors elaborated the acquired data, reaching percentages of discrimination of the cheeses concerning milk thermal treatment > 93%.

The results of this work in the detection of milk thermal treatment in Fiore Sardo PDO cheese were very promising. Even if the result obtained by the model that considered the three categories of samples was not highly accurate, the other three models built to discriminate raw-milk cheese samples from thermized samples (TC, LTC, or HTC, respectively) correctly identified the milk thermal treatment of about 90% of the cheese samples. A strategy of using the discriminant models on Fiore Sardo cheeses could be to predict the category of an unknown sample by simultaneously using these three models and comparing the results. In fact, the total number of misclassified samples for the three models was 19 out of 105 samples; a total of 15 of them were assigned to the wrong category only by one of these models, whereas the other two models correctly detected the thermal treatment category (Table 3). After assessing the presence of thermal treatment, the spectrum of a thermized sample could even be read using the LTC vs. HTC model to identify the temperature of thermization.

This work demonstrated that NIR spectroscopy and chemometrics were able to identify mild milk thermal treatments in Fiore Sardo cheese and that this type of method is a valuable tool to detect fraud caused by the heating of milk before the cheese-making process. To gain the certainty of having reliable and stable models, the calibration dataset should be increased to include samples of different geographical areas and periods (the main variation causes of milk composition) to make the models applicable to cheeses of different origin. The variability in the lactation stage of sheep (January to June) was considered in this work but the variability of milk over years and over different geographical areas of the region is higher than that related to a single lactation stage, making it more difficult to identify raw and thermized milk. In that case, it is likely that a lower amount of spectral regions should be selected to avoid those regions related to other milk variability. Having expanded the models, they should be checked on a validation set of samples not considered in the calibration steps, as well as testing commercial samples produced in different areas of the region. In this way, the reliability of the models over time would be better assessed.

The importance of the method developed in this work lies in the simplicity of its application to a fraud control system, measuring samples in a fast way without the use of chemicals. NIR instrumentation only requires the use of grated cheese, recording its spectrum in a few seconds; it is therefore suitable as a fast screening method for the detection of the mild thermal treatment of the milk in Fiore Sardo PDO cheese samples. Samples that after screening, result in doubts relating to their origin could also be checked with other techniques such as enzymatic methods to assess the presence of the thermization of milk.

## 5. Conclusions

The aim of this study was to use NIR spectroscopy combined with chemometrics to determine the thermal treatment of the milk processed to obtain Fiore Sardo PDO cheese. The PDO specification of this cheese requires the use of raw milk, and the thermal treatment of milk is one of the main types of fraud concerning this type of cheese. The NIR spectra of Fiore Sardo cheeses produced with raw milk (38 °C), low-thermized milk (57 °C, 30 s), and high-thermized milk (68 °C, 30 s) were subjected to LDA and to a selection of wavelengths carried out using genetic algorithms. Different spectral pretreatments were evaluated in the building of the discriminant models. The results obtained were very promising in discriminating cheeses made from thermized versus raw milk (accuracies of discrimination close to 90%). The method can be further developed by increasing the calibration step with samples that include significant variability in Sarda sheep milk, as well as by assessing the models through an external types of validation using unknown samples.

## Figures and Tables

**Table 1:** Results obtained in the building of the LDA models for all the sample categories to be discriminated, considering all the spectral treatments and the three variable selection options (best results in bold).

Categories to Be Discriminated	Spectral Pretreatment	Accuracy (%)	Accuracy (%)	Accuracy (%)
		LDA Model Using the Wavelengths Selected on the Entire Spectra (By Averaging over 4 Adjacent Variables)	LDA Model Using the Wavelengths Selected on Two Portions of the Spectra (By Averaging over 2 Adjacent Variables)	LDA Model Using the Wavelengths Selected on Four Portions of the Spectra (Without Averaging Variables)
RC vs. LTC vs. HTC	None	47	57	63
	None + 1st derivative	53	58	59
	None + 2nd derivative	43	51	67
	SNV	57	56	60
	SNV + 1st derivative	46	63	74
	SNV + 2nd derivative	53	51	**76**
	MSC	41	60	64
	MSC + 1st derivative	48	54	65
	MSC + 2nd derivative	45	67	65
RC vs. LTC	None	74	76	73
	None + 1st derivative	54	70	76
	None + 2nd derivative	53	67	**93**
	SNV	70	73	77
	SNV + 1st derivative	71	59	81
	SNV + 2nd derivative	79	79	81
	MSC	56	64	77
	MSC + 1st derivative	71	79	84
	MSC + 2nd derivative	80	80	81
RC vs. HTC	None	67	71	81
	None + 1st derivative	70	77	90
	None + 2nd derivative	77	81	83
	SNV	71	67	80
	SNV + 1st derivative	81	89	89
	SNV + 2nd derivative	90	86	87
	MSC	70	70	80
	MSC + 1st derivative	64	**91**	84
	MSC + 2nd derivative	70	83	**91**
RC vs. TC	None	66	73	74
	None + 1st derivative	64	72	80
	None + 2nd derivative	54	65	**89**
	SNV	61	77	79
	SNV + 1st derivative	73	71	79
	SNV + 2nd derivative	71	68	84
	MSC	59	75	73
	MSC + 1st derivative	74	78	80
	MSC + 2nd derivative	67	76	81
LTC vs. HTC	None	66	71	69
	None + 1st derivative	81	71	83
	None + 2nd derivative	86	73	84
	SNV	61	73	80
	SNV + 1st derivative	73	74	79
	SNV + 2nd derivative	64	86	86
	MSC	66	73	76
	MSC + 1st derivative	67	77	84
	MSC + 2nd derivative	63	**90**	84

RC = raw milk cheese samples; LTC = low-thermized-milk cheese samples; HTC = high-thermized-milk cheese samples; TC = thermized-milk cheese samples. SNV = standard normal variate; MSC = multiplicative scatter correction. The best results for each type of comparison are shown in bold.

**Table 2:** Confusion matrixes of the best LDA models of each type of comparison and the corresponding selected spectral regions.

	**RC**	**LTC**		**HTC**	**Sensitivity**	**Specificity**	**F1 Score**	**Accuracy (%)**	**Selected Spectral Regions (2nd Derivative SNV Spectra)**
RC	27	5		3	0.771	0.914	0.794		1256–1258 nm, 1306–1308 nm, 1378–1380 nm, 1466–1470 nm, 1474 nm, 1676 nm, 1680–1688 nm, 1832 nm, 2356–2360 nm
LTC	4	25		6	0.714	0.857	0.714	76	
HTC	2	5		28	0.800	0.871	0.778		

	**RC**		**LTC**		**Sensitivity**	**Specificity**	**F1 Score**	**Accuracy (%)**	**Selected Spectral Regions (2nd Derivative Spectra)**
RC	34		1		0.971	0.886	0.931	93	1136 nm, 1198 nm, 1254–1262 nm, 1306 nm, 1322–1324 nm, 1432 nm, 1436–1438 nm, 1470–1472 nm, 1594–1598 nm, 1930 nm, 2260–2264 nm, 2362 nm, 2376–2380 nm, 2408–2414 nm, 2460–2468 nm
LTC	4		31		0.886	0.971	0.925		

	**RC**		**HTC**		**Sensitivity**	**Specificity**	**F1 Score**	**Accuracy (%)**	**Selected Spectral Regions (2nd Derivative MSC Spectra)**
RC	32		3		0.914	0.914	0.914	91	1574–1576 nm, 1606–1612 nm, 1678–1690 nm, 1840–1844 nm, 2152–2156 nm, 2262–2266 nm
HTC	3		32		0.914	0.914	0.914		

	**RC**		**TC**		**Sensitivity**	**Specificity**	**F1 Score**	**Accuracy (%)**	**Selected Spectral Regions (2nd Derivative Spectra)**
RC	31		4		0.886	0.886	0.838	89	1106 nm, 1132–1134 nm, 1256–1260 nm, 1472 nm, 1540 nm, 1596–1598 nm, 1618–1622 nm, 1838–1840 nm, 1950 nm, 2020–2024 nm, 2158 nm, 2262–2264 nm, 2376–2378 nm
TC	8		62		0.886	0.886	0.912		

	**LTC**		**HTC**		**Sensitivity**	**Specificity**	**F1 Score**	**Accuracy (%)**	**Selected Spectral Regions (2nd Derivative MSC Spectra)**
LTC	31		4		0.886	0.914	0.899	90	1472–1476 nm, 1568–1570 nm, 1612–1614 nm, 1672–1686 nm, 1704–1706 nm, 2436–2438 nm
HTC	3		32		0.914	0.886	0.901		

**Table 3:** Misclassified samples of the best LDA models of each type of comparison (numbers correspond to the position of the samples in the dataset).

Category	Models				
	RC vs. LTC vs. HTC	RC vs. TC	RC vs. LTC	RC vs. HTC	LTC vs. HTC
RC				3	
	4				4
			7		
	11	11			11
		15			
	16				16
	20				20
	21	21			21
	25	25		25	25
	27				27
	31			31	31
LTC		38			
					41
	42				
	45		45		
	48	48	48		
	53				
	55	55	55		
	57				57
	60				
	61				
	62				62
					64
			65		
	67				
		70			
HTC		71		71	71
				80	
	81	81			
		82			
					84
		85			
	86			86	
	94				
	98				
	100				100
	101				
	105				
		71		71	71

## Data Availability

The raw data supporting the conclusions of this article will be made available by the authors on request.

## Abbreviations

The following abbreviations are used in this manuscript:

PDO	Protected designation of origin
NIR	Near-infrared
ALP	Alkaline phosphatase
GGT	γ-glutamyltransferase
NMR	Nuclear magnetic resonance
MRI	Magnetic resonance imaging
GA	Genetic algorithm
FT-MIR	Fourier transform mid infrared
RM	Raw milk
LTM	Low-thermized milk
HTM	High-thermized milk
RC	Raw-milk cheeses
LTC	Low-thermized-milk cheeses
HTC	High-thermized-milk cheeses
PCA	Principal component analysis
LDA	Linear discriminant analysis
SNV	Standard normal variate
MSC	Multiplicative scatter correction
CV	Cross validation
